# Prevalence of Myocardial Fibrosis in Intensive Endurance Training Athletes: A Systematic Review and Meta-Analysis

**DOI:** 10.3389/fcvm.2020.585692

**Published:** 2020-09-25

**Authors:** Cheng-Duo Zhang, Shun-Lin Xu, Xin-Yu Wang, Li-Yuan Tao, Wei Zhao, Wei Gao

**Affiliations:** ^1^National Health Commission Key Laboratory of Cardiovascular Molecular Biology and Regulatory Peptides, Key Laboratory of Molecular Cardiovascular Science, Ministry of Education, Beijing Key Laboratory of Cardiovascular Receptors Research, Department of Cardiology and Institute of Vascular Medicine, Peking University Third Hospital, Beijing, China; ^2^Department of Epidemiology, Peking University Third Hospital, Beijing, China

**Keywords:** cardiac magnetic resonance imaging, gadolinium enhancement, endurance training, myocardial fibrosis, athletes heart

## Abstract

**Objective:** To review the published literature reporting on the incidence of myocardial fibrosis (MF) in high-intensity endurance athletes measured by late gadolinium enhancement (LGE) with cardiac magnetic resonance imaging (CMR).

**Methods:** Five databases (PubMed, Cochrane Controlled Trials Register, EMBASE, Web of Science, and SPORTDiscus) were searched to obtain case cohort studies published before November 10, 2019. From 96 abstracts or reports extracted, 18 full-text articles were reviewed. The incidence of LGE was reported as outcome measures. Subgroup analysis was performed by age (under or above 50 years). Pooled estimates were obtained using a fixed-effects model.

**Results:** After a full-text assessment, 12 studies involving 1,359 participants were included for analysis. Among them, 163/772 participants in the endurance athletes group showed LGE positive, compared with 19/587 participants in the comparison group. The results of the meta-analysis suggested that the prevalence of LGE was higher in the athletes group with long-term endurance exercise (OR 7.20;95%CI: 4.51–11.49). In addition, the same conclusion was drawn after the stratification of age.

**Conclusions:** The available evidence demonstrates that high-intensity endurance athletes is associated with an increased incidence of LGE positive.

## Introduction

Moderate intensity exercise is associated with a risk of cardiovascular disease that is one third less (1). Some studies indicate that exercise-related cardiovascular benefits still seem to be found in extreme endurance athletes compared to the general population (2).

When the body is engaged in high-intensity endurance exercise, the metabolic state is completely different from the quiet state, characterized by increased catecholamine release, heart rate, oxygen demand and free fatty acid metabolism, coronary artery contraction, decreased coronary perfusion, lactic acidosis and so on (3). Long-term high-intensity endurance training can have a positive impact on the heart. For instance, athletes' hearts can demonstrate enlarged ventricular cavity, left ventricular (LV) hypertrophy, increased myocardial mass and cardiac output, and decreased resting heart rate. These changes allow one's body to exercise continuously for hours in an aerobic state (4).

However, high-intensity endurance training could also have adverse potential effects on the heart, such as electrophysiological abnormalities (5) and elevated cardiac markers, including creatine kinase-MB, troponin T and NT-pro brain natriuretic peptide (2). Braber et al. (6) indicated that high-intensity endurance training maybe associated with accelerated progression of coronary atherosclerosis and that coronary heart disease remained the leading cause of death for high-intensity endurance athletes over the age of 35.

MF is a condition involving the significant increase of collagen volume in myocardial tissue, which results from myocardial tissue injury caused by myocardial ischemia (hypoxia), inflammation and hypertension overload (7). Specific types of MF are associated with ventricular arrhythmia and adverse cardiac events. MF has been detected in some asymptomatic endurance athletes by the application of CMR with LGE in a non-invasive way.

By summarizing the available data on the prevalence of MF in physically active individuals, van de Schoor et al. (7) attempted to identify predictors and potential mechanisms of MF development and its clinical implications. However, only two studies were listed to compare MF prevalence between endurance athletes and matched non-exercise comparison groups.

Therefore, we aimed to carry out a meta-analysis of relevant literature and analyzed the incidence of LGE positive measured by CMR between high-intensity endurance athletes and inactive controls of similar age and gender.

## Methods

### Protocol and Registration

This study protocol was registered with PROSPERO and approved with registration number (CRD42020161240).

### Eligibility Criteria

High-intensity endurance athletes and age- and sex-matched physically inactive controls were settled as subjects. Both groups were required to be healthy and free of cardiopulmonary disease. High-intensity endurance athletes were defined as having certain competition experience or a long period of regular high intensity endurance training. The inactive comparison group was defined as not participating in regular exercise or exercising <3 h per week. Case cohort study with MF measured by CMR will meet the inclusion criteria.

Articles were excluded from the review if they were non-English literature, repeatedly published literature, with incomplete data to determine grouping or unable to calculate OR value, or not measured by CMR to determine MF.

### Data Source and Search Strategy

A systematic search of relevant English-language studies published up to November 10, 2019 was performed in the following databases: PubMed, Cochrane Controlled Trials Register, EMBASE, Web of Science, and SPORTDiscus. Key medical subject heading terms and keywords related to marathon (endurance training, intensive/extreme/strenuous/exhaustive endurance training) and lated gadolinium enhancement (LGE, lated enhancement, delayed gadolinium enhancement, gadolinium) were used. We executed our search and reported our findings according to the guidelines of the Meta-Analysis of Observational Studies in Epidemiology Group.

### Study Selection and Data Extraction

Two reviewers independently conducted the literature search and extracted data of relevant articles. The title and abstract of potentially relevant studies and review articles were screened for appropriateness before retrieval of the full articles. Endnote software was used for literature review and screening. Extracted items of the study included: author, year, country, design, sample size, number of athletes detected with MF, characteristic of endurance athletes and inactive controls, age, and gender. Outcomes abstracted included the result of LGE assay for MF as a dichotomous variable. Disagreements between two reviewers were resolved by consensus.

### Study Quality Assessment

To evaluate the methodological quality of the selected studies, we used the Newcastle-Ottawa quality assessment scale (NOS). The NOS allocates points for three domains: selection (four items), comparability (two items), and outcome for cohort studies or exposure for case-control studies (three items). Each item was given one star if addressed. Studies with cumulative score >6 stars, 6 stars, and <6 stars were considered to be of high quality, moderate quality, and low quality, respectively. Any disagreement in study quality assessment was resolved by discussion with the third author (Li-Yuan Tao) to reach a consensus.

### Data Synthesis

The statistical analysis was performed using Review Manager (Version 5.3, USA). Heterogeneity was assessed by *Q* test and *I*^2^ statistic. A *P* < 0.1 or an *I*^2^ statistic of ≥50% was regarded as apparent heterogeneity among research results. When there was heterogeneity, a random-effects model was adopted for combined analysis. Otherwise, a fixed-effects model was used. To rule out the possibility that any study had an excessive impact on the results, we performed the sensitivity analysis, systematically excluding each study at a time and then re-running the analysis to assess the change of effect size. All *P*-values were 2-sided and considered significant when <0.05.

## Results

### Research Characteristics and Quality Evaluation

**Figure 1:**
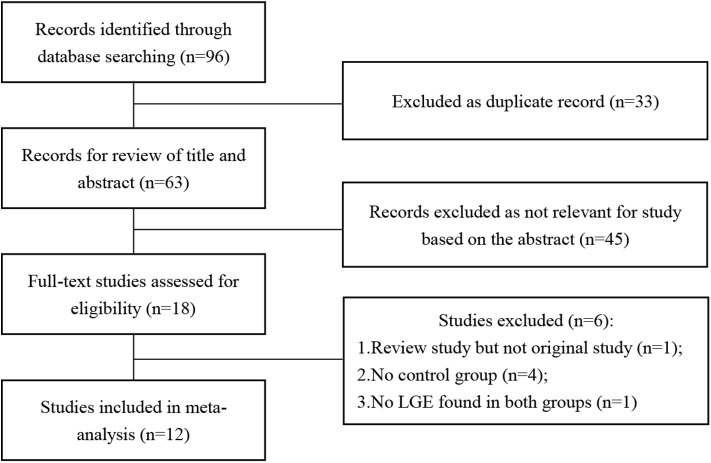
Flow chart of literature selection.

The results of the literature search are displayed in Figure 1. Finally, 12 eligible studies using CMR to investigate the prevalence of LGE in high-intensity endurance athletes and matched physically inactive controls were included, with a total of 1,359 participants.

**Figure 2:**
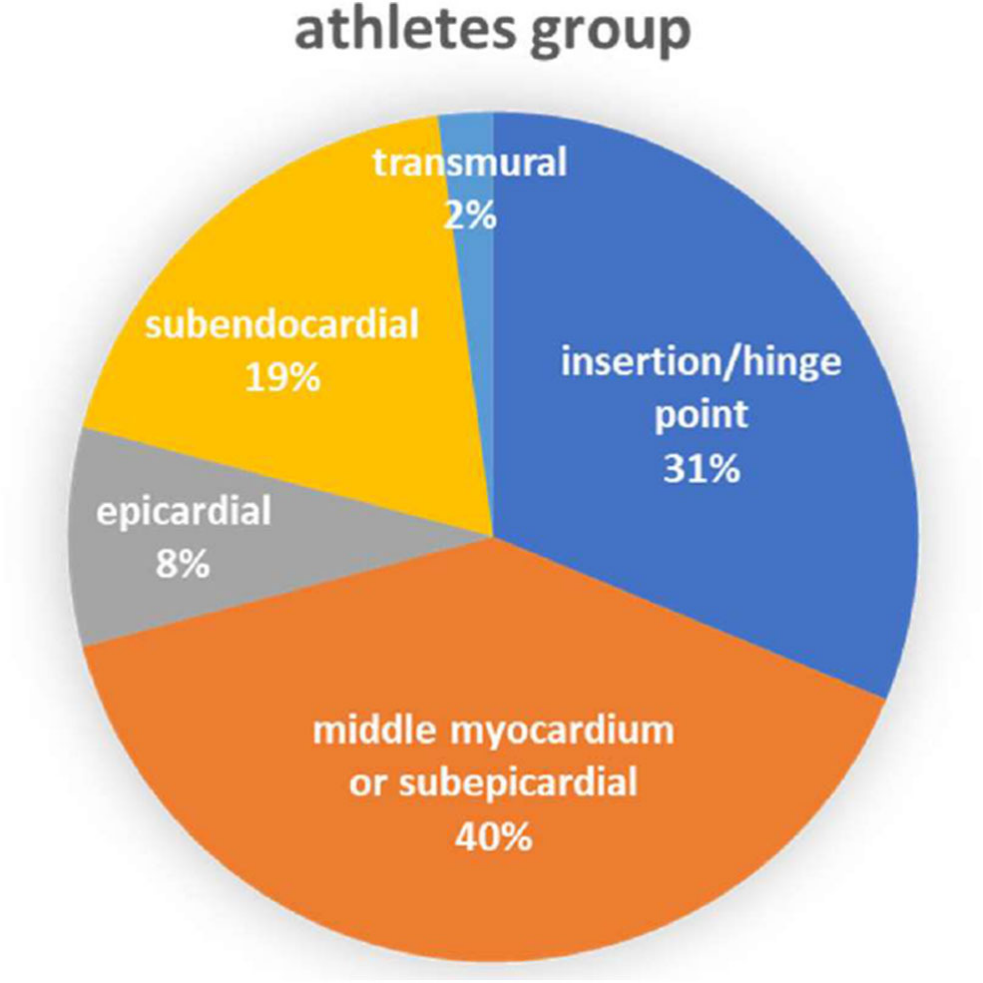
Distribution of LGE in athletes group.

Table 1 summarizes the characteristics of the included articles. The LGE distribution was not recorded in 3 articles and 1 LGE positive athlete's message was unclear. Totally, there are 48 LGE positive athletes recorded detailedly in 9 researches (Figure 2) while 3 LGE positive are all at the hinge-point area in the comparison group. All those 12 studies were of case-cohort design and conducted between 2009 and 2019. In terms of geographic area, 3 were conducted in Germany, 3 in Britain, 3 in Spain, 1 in Canada, 1 in the USA, and 1 in Poland. According to the NOS criteria, 10 studies were of high quality, and the quality of 2 studies was inadequate due to incomplete data. However, since those 2 articles were published in authoritative journals, they were also included. RevMan automatically adds 0.5 when the number of events in the observation group or comparison group is 0. However, when the number of events in both groups is zero, it is not possible to perform statistical analysis. Thus, we excluded one study in which the incidence of MF was 0 in both groups.

**Table 1:** Prevalence and patterns of myocardial fibrosis in athletic populations using CMR.

**References**	**Country**	**Study population**	**Exercise exposure**	**Age (y), mean ± SD**	**Sex**	**Prevalence of MF**	**Pattern/location of MF**
Breuckmann et al. (8)	German	Marathon runners (*n* = 102) Age-matched control (*n* = 102)	5 full-distance marathons during the past 3 years No exceptional endurance sports activity	57 ± 6	100% male	Athletes: 12/102 Controls: 4/102	Athletes: 5 CAD pattern (involving the subendocardial layer and partial transmural spreading); 7 non-CAD pattern Controls: 2 CAD pattern; 2 non-CAD pattern
Wilson et al. (9)	UK	Lifelong veteran endurance athletes (marathon/triathlons) (*n* = 12) Veteran sedentary controls (*n* = 20)	Competitive exercise training for many years No exercise training	57 ± 6 60 ± 5	100% male	Athletes: 6/12 Controls: 0/20	Athletes: 1 CAD pattern (septal and lateral wall); 5 non-CAD pattern (epicardial lateral wall; basal and mid insertion point; inferior insertion point mid and apical; insertion point inferior mid/apical; inferior insertion point)
Altaha et al. (10)	Canada	Sub-elite athletes (running/cycling/triathlons) (*n* = 32) Recreational fitness participants (*n* = 4)	>10 years of exercise <3 h exercise/week	55 ± 5.6	69.4% male	Athletes: 4/32 Controls: 1/4	All appeared as minor LV LGE at the inferior RV hinge-point; no significant ischemic or non-ischemic LV LGE
Bohm et al. (11)	German	Elite master endurance athletes (marathon/triathlons/rowing/cyclist) (*n* = 33) Age-, height-, and weight-matched individuals (*n* = 33)	Training history of ≥10 years and ≥10 h per week No exercise training	47 ± 8 46 ± 9	100% male	Athletes: 1/33 Controls: 0/33	Athletes: 1 non-ischemic LV LGE; subepicardially in the LV posteroinferior region on the short-axis view
Sanchis-Gomar et al. (12)	Spain	Sub-elite and elite athletes (cyclists, runners) (*n* = 10) Age-matched controls (*n* = 5)	Experience in competition for many years Never participated regularly in strenuous endurance; exercise performing <3 structured weekly training sessions	40–70	100% male	Athletes: 2/10 Controls: 0/5	Athletes: 2 non-coronary pattern by LGE; LV lateral wall and basal segment of the inferolateral LV wall
Abdullah et al. (13)	USA	Masters athletes (triathlons/runners) (*n* = 21) Age- and gender-matched non-athletic control(*n* = 71)	>3 h training/week at least during the last 25 years and regular competitions <3 h training/week at least during the last 25 years	69.2	73% male	Athletes: 0/21 Controls: 1/71	Controls:1 small focus of LGE seen in the intraventricular septum close to the inferior right ventricular insertion point
Merghani et al. (14)	UK	Master athletes (cyclists/runners) (*n* = 152) Controls with similar age, sex, low Framingham 10 year CAD risk score (*n* = 92)	≥10 miles running or ≥ 30 miles cycling per week for at least 10 years, completed at least 10 competition Engaged in exercise in accordance with the physical activity recommendations for health	54.4 ± 8.5	70% male	Athletes: 16/152 Controls: 0/92	Athletes: 7 male athletes showed sub-endocardial LGE (consistent with myocardial infarction); 5 had a midmyocardial distribution and 3 had an epicardial distribution; 1 female athlete showed sub-endocardial LGE
Zaidi et al. (15)	UK	Master endurance athletes (*n* = 170) Age- and gender-matched non-athletic control (*n* = 130)	Details were unavailable for both groups	54.4 ± 8.5	71.2% male	Athletes: 69/170 Controls: 8/130	Details were unavailable
Domenech Ximenos et al. (16)	Spain	Highly trained endurance athletes (*n* = 93) Age- and gender-matched controls (*n* = 72)	>12 h training/week at least during the last 5 years Details were unavailable	35 ± 5.1	52% male	Athletes: 33/93 Controls: 4/72	Always confined to the hinge point; more details were unavailable
Pujadas et al. (17)	Spain	Healthy endurance Runners (*n* = 34) Age and body surface area matched controls (*n* = 12)	10 years of training and with marathon times below 3 h and 15 min No exercise training	48.1 ± 7.4	100% male	Athletes: 3/34 Controls: 0/12	Athletes: non-ischemic LGE pattern was noted: mesocardial in septal-apical wall, subepicardial in the inferior apical wall and mesocardial in the lateral wall
Tahir et al. (18)	Germany	Triathletes (*n* = 83) Age- and sex-matched controls (*n* = 36)	>10 training hours per week or regularly participated in triathlons in the previous 3 years <3 h exercise per week	43 ± 10	65% male	Athletes: 9/83 Controls: 0/36	Athletes: 5 triathletes had predominantly subepicardial LGE locations with a thin pericardial gap; 2 athletes had LGE located at the posterior right ventricle insertion point; 1 had an almost transmural LGE, but the sub-endocardial area was spared
Małek et al. (19)	Poland	Healthy ultra-marathon runners (*n* = 30) Age- and sex-matched controls (*n* = 10)	Median 9 years of running with frequent competitions Not engaged in any regular activities	40.9 ± 6.6	100% male	Athletes: 8/30 Controls: 1/10	Athletes: no cases of ischemic (sub-endocardial) LGE; 5 with a spotty-shaped, focal, midwall lower insertion point fibrosis; 3 with mid-wall or subepicardial, linear, very limited LGE Controls: 1 with spotty-shaped, focal, midwall lower insertion point fibrosis; no cases of ischemic (sub-endocardial) LGE

*CMR, cardiac magnetic resonance imaging; CAD, coronary artery disease; LV, left ventricular; LGE, late gadolinium enhancement; RV, right ventricular*.

**Figure 3:**
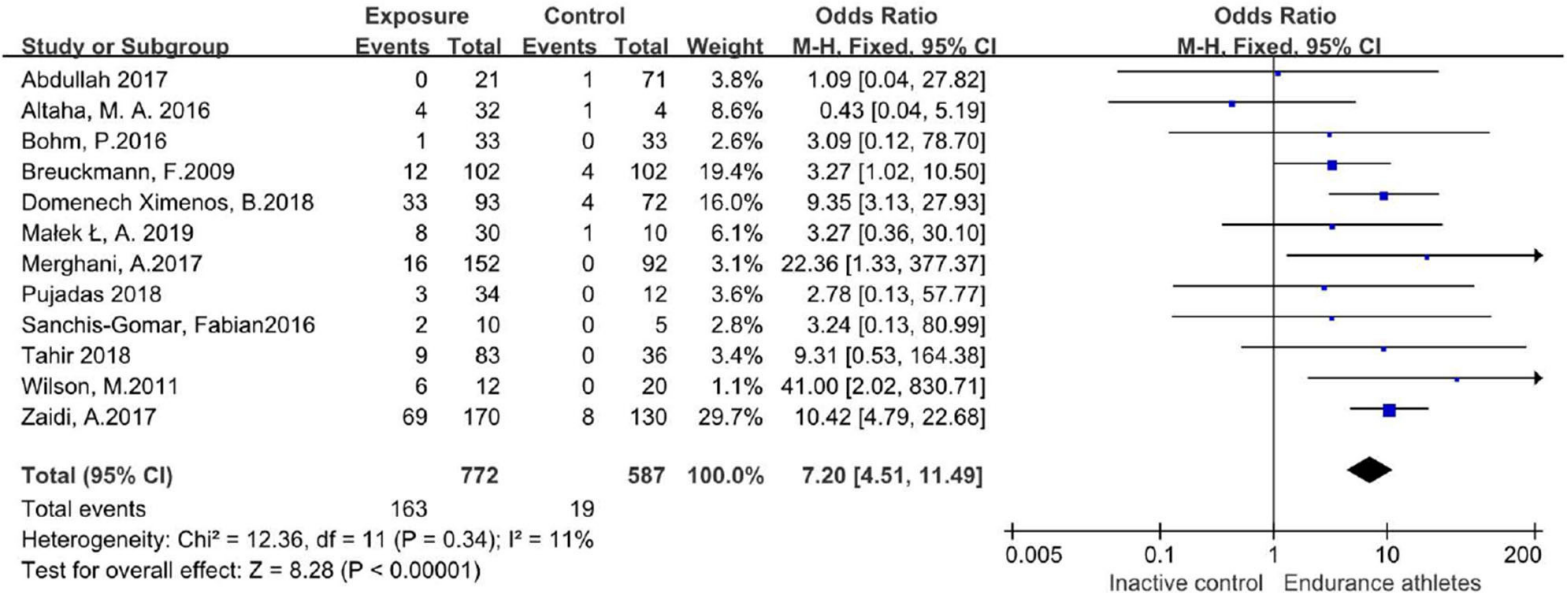
Forest plot of total incidence of LGE.

In those studies, 163/772 participants in the endurance athletes group showed LGE positive, compared with 19/587 participants in the comparison group. There was no heterogeneity during each study period, so a fixed-effects model was adopted. The meta-analysis showed an increased risk of the endurance athletes group by 7.2-fold with LGE compared with the comparison group (OR 7.20;95%CI: 4.51–11.49; *I*^2^ = 11%; *P* = 0.34) (Figure 3).

### Sensitivity Analysis

**Figure 4:**
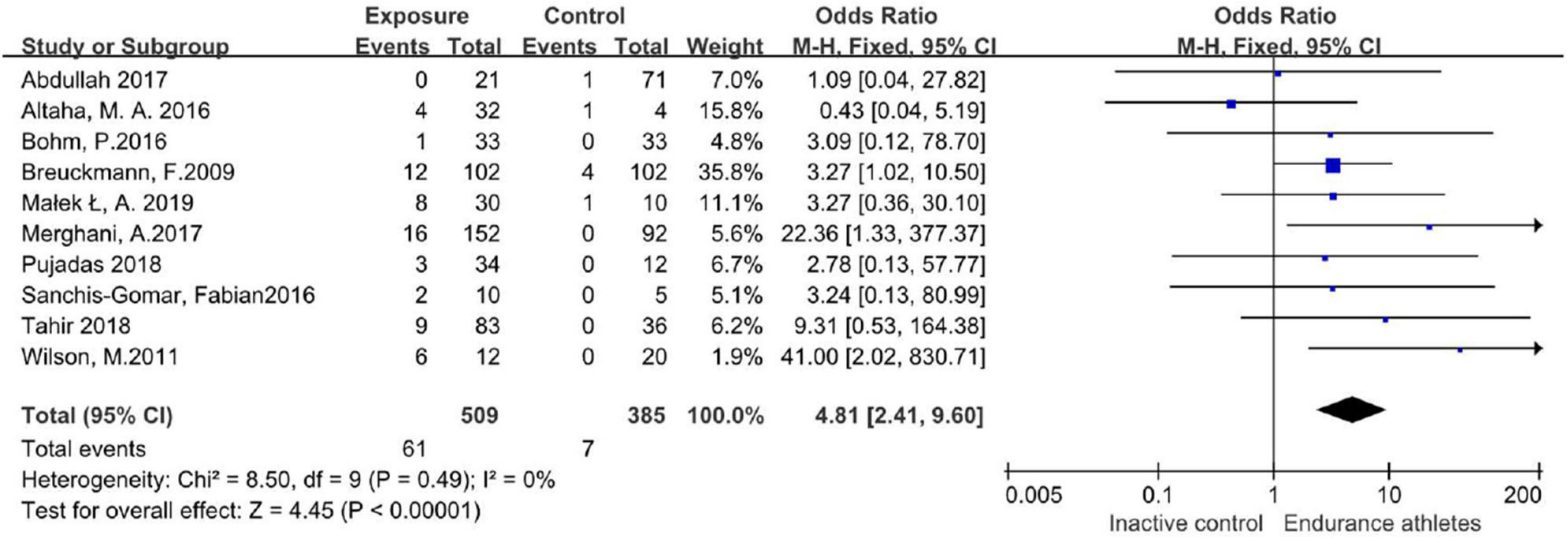
Forest plot of incidence of LGE after excluding 2 low-quality researches.

No particular study excessively influenced the results. Even if two studies with missing data were excluded, the conclusion remained unchanged (Figure 4).

### Subgroup Effect Analysis

**Figure 5:**
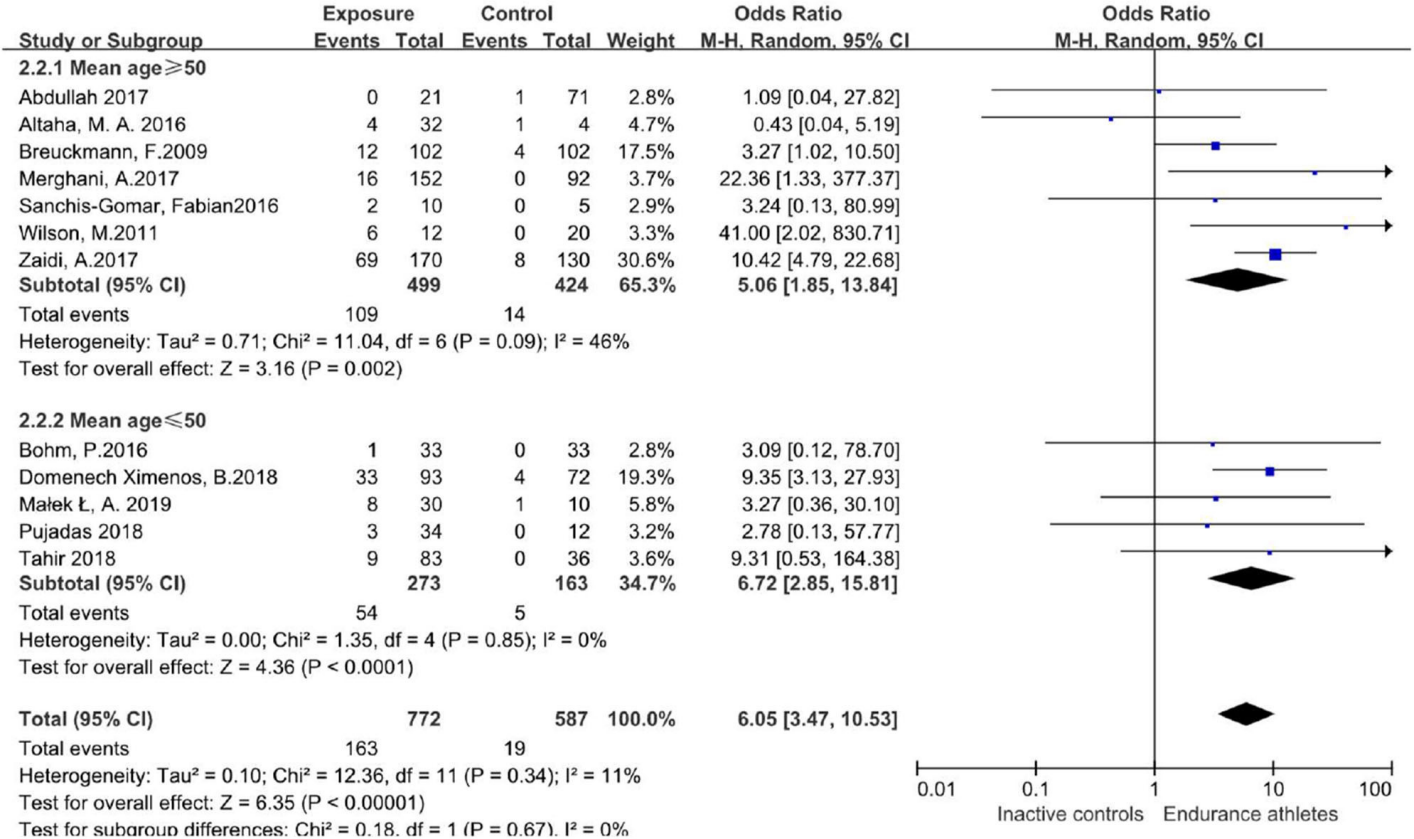
Odds ratio of LGE in both groups, stratified by age.

We further performed meta-regression with covariates including age to quantify the heterogeneity. After stratification by age (mean age ≥ 50 years, mean age ≤ 50 years), the prevalence of LGE was still different between athletes and the comparison group with no significant heterogeneity (Figure 5).

### Publication Bias Analysis

**Figure 6:**
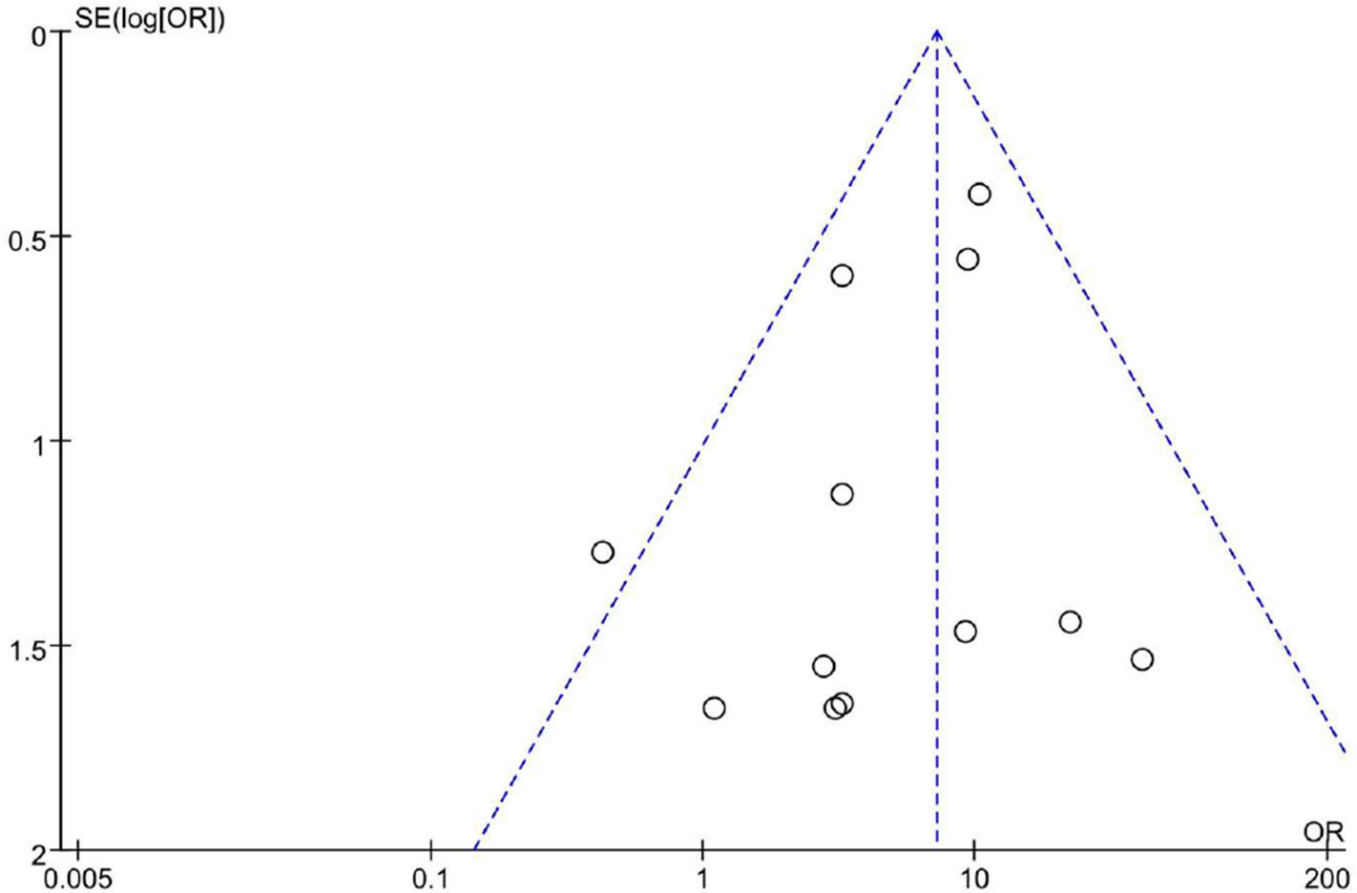
Funnel plot of incidence of LGE.

Totally we just included 12 studies. As shown in Figure 6, the funnel plot revealed a proper symmetric distribution of the included studies on both sides, suggesting no obvious publication bias.

## Discussion

Several studies have explored whether elevated markers of myocardial injury after the competition are related to injury by performing cardiac magnetic examinations before and after endurance activities. However, little newly emerged LGE was found after exercise, suggesting that the elevation of markers was not induced by myocardial necrosis (20–22). However, repetitive exposure to high-intensity endurance exercise can induce cardiac microinjury and increased ventricular wall tension during exercise, which further leads to MF development after lifelong exercise training, as observed in veteran athletes (9).

Since MF has been confirmed in endurance athletes, the relationship between lifelong endurance exercise and MF development is unclear.

This study represents an analysis of the currently reported LGE data among high-intensity endurance athletes and age- and sex-matched physically inactive controls. The results showed that there was a statistically significant difference in the incidence of LGE between athletes and the comparison group. Turkbey et al. conducted a large sample cohort study on non-athletes in the United States (mean [SD] age, 68[±9] years, 52% men) and found that 7.9% (146/1,840) of the subjects had MF (23). In our study, participants of the 12 enrolled researches around the world were mostly middle-aged men. The incidence of LGE was 3.23% (19/587) in the comparison group and 21.1% (163/772) in the athlete group, which was, respectively, lower and significantly higher than the incidence found in Turkbey's study.

A coronary artery disease (CAD) pattern of LGE is the delayed enhancement of progression from endocardium to epicardium located in the coronary blood supply area. In a few of those athlete subjects, CMR-detected LGE was consistent with previous infarction and appeared to be associated with increased risk of cardiovascular events (7). According to Van de Schoor's research, the LGE found among middle-aged and older athletes was primarily confined to the interventricular septum, often near the hinge points between right ventricular (RV) and the septum. This pattern appears to be more common in long-term endurance athletes and may be the result of repeated myocardial micro-injury or the expansion of RV (7, 24). Non-ischemic LV LGE with a stria morphological pattern and subepicardial/midmyocardial distribution as reported in listed researches (Table 1) is not a benign finding in asymptomatic athletes and may be associated with life-threatening arrhythmias or sudden death. This LGE pattern might be the result of prior silent myocarditis or cardiomyopathies, but the exact pathophysiology and prognostic significance in athletes are still debated (25, 26). Focal LGE could be found mostly at the middle myocardium/subepicardial of LV and insertion/hinge points of RV as we summarized in the meta-analysis.

LGE positive is related to inflammation in acute myocarditis and fibrosis in the chronic stage. Ischemia tends to occur in subendocardial while inflammatory reaction is prone to occur in subepicardial due to different degree of blood supply. LGE is mainly located under endocardium or transmural spreading during myocardial ischemia while distributed under epicardium or middle myocardium in myocarditis (27). None of the people included in this Meta-analysis are in acute exercise stage or unhealthy state. LGE positive is more likely to be associated with fibrosis than acute inflammation. CAD and certain non-ischemic LV pattern of LGE suggests the need for pre-participation physical evaluation (PPE). Patients with myocarditis need to rest for 3–6 months and only after a comprehensive evaluation can they return to physical training (28).

Cardiac fibrosis caused by intense exercise may be a reactive phase of MF (that is, abnormal growth of extracellular matrix and deposition around cells or blood vessels). Studies have confirmed that high-intensity endurance exercise can lead to the occurrence of MF in mice, and importantly, the authors documented that the cessation of endurance training was able to arrest and even reverse this pathological process (29). However, similar pathophysiological processes have not been confirmed in human trials.

When athletes participate in high-intensity endurance races, especially marathon races, sudden cardiac death (SCD) events are often reported and attract wide attention of the society, even though the absolute SCD risk caused by marathon may not be as high as we thought (the incidence rate is <1/100,000) (30–34). Therefore, the correlation between LGE and adverse cardiovascular adverse events remains to be further confirmed.

Recently, the native T1 mapping technique using CMR imaging has shown promise in evaluating myocardial fibrosis. Some studies have shown that diffuse myocardial fibrosis evaluated by histology correlates to native T1 values and have demonstrated that T1 mapping can be comparable to LGE imaging for detecting myocardial scar extensions. The cardiac MRI T1 mapping technique is emerging as a valuable non-invasive method that may enable the quantification of the myocardial extracellular volume fraction (ECV) for the detection of diffuse myocardial fibrosis. A few studies on T1 mapping in athletes and non-athletes have been carried out, but the conclusions are still inconsistent, so more research is needed to find out (35–37).

## Limitation

This study has several limitations. First, in this rather new research area, only studies written in English were included in the review. Relevant articles written in other languages were possibly missed. Second, a rather small number of studies satisfy the inclusion criteria, and all of them are case-cohort studies. Therefore, more prospective clinical trials in the future can improve the accuracy of the conclusions. Third, the included studies only covered Europe and North America, and some literature did not provide enough detailed daily training intensity, so the representativeness was limited. Thus, it should be cautious to extrapolate our findings to other populations such as amateur runners or people with regular aerobic training.

Finally, due to research design limitations, no confounding factors were taken into account. Hence, the reported odds ratio should be treated with care as it may change when adjusted for sex, cardiovascular risk factors, blood pressure, etc. Because exact pathophysiology and consequences of LGE in athletes are still debated, these athletes require additional follow-up to determine the significance of this finding.

## Conclusion

According to the current available data, there is a statistically significant increase in the incidence of LGE positive, which refers to myocardial fibrosis, associated with intensive endurance training. Further studies are needed to investigate the clinical significance of LGE positive in endurance athletes.

## Data Availability Statement

All datasets generated for this study are included in the article/supplementary material.

## Author Contributions

While conducting this review, C-DZ played a role in overseeing data collection and editing this paper. S-LX played a role in overseeing data collection and literature review update. L-YT played a role in data analysis. X-YW played roles in interpretation and analysis of CMR results. WZ and WG played a role in conceiving, designing, and prepared the initial draft of the manuscript. All authors have read and approved the final version of the manuscript, and agreed with the order of presentation of the authors.

## Conflict of Interest

The authors declare that the research was conducted in the absence of any commercial or financial relationships that could be construed as a potential conflict of interest.
